# Perceptual metrics for odorants: Learning from non-expert similarity feedback using machine learning

**DOI:** 10.1371/journal.pone.0291767

**Published:** 2023-11-08

**Authors:** Priyadarshini Kumari, Tarek Besold, Michael Spranger

**Affiliations:** 1 Sony AI, Sunnyvale, California, United States of America; 2 Sony AI, Barcelona, Spain; 3 Sony AI, Tokyo, Japan; BML Munjal University, INDIA

## Abstract

Defining perceptual similarity metrics for odorant comparisons is crucial to understanding the mechanism of olfactory perception. Current methods in olfaction rely on molecular physicochemical features or discrete verbal descriptors (floral, burnt, etc.) to approximate perceptual (dis)similarity between odorants. However, structural or verbal descriptors alone are limited in modeling complex nuances of odor perception. While structural features inadequately characterize odor perception, language-based discrete descriptors lack the granularity needed to model a continuous perception space. We introduce data-driven approaches to perceptual metrics learning (PMeL) based on two key insights: a) by combining physicochemical features with the user’s perceptual feedback, we can leverage both structural and perceptual attributes of odors to define dissimilarity, and b) instead of discrete labels, user’s perceptual feedback can be gathered as relative similarity comparisons, such as “Does molecule-A smell more like molecule-B, or molecule-C?” These triplet comparisons are easier even for non-experts users and offer a more effective representation of the continuous perception space. Experimental results on several defined tasks show the effectiveness of our approach in evaluating perceptual dissimilarity between odorants. Finally, we investigate how closely our model, trained on non-expert feedback, aligns with the expert’s similarity judgments. Our effort aims to reduce reliance on expert annotations.

## Introduction

All five basic senses—sight, touch, smell, hearing, and taste- contribute to enriching our daily lives. For example, the deliciousness of food that we enjoy every day is a result of all five senses. In fact, contrary to common beliefs, several studies assert that taste perception is stimulated more by olfactory receptors than by gustatory receptors [1, 2]. The recent surge in artificial intelligence (AI) makes an effort toward emulating human perceptual intelligence in machines. While our knowledge about machine vision and hearing has grown rapidly over the past few decades, the mechanism of *chemical* senses (taste and smell) remains poorly understood.

A century ago, Graham Bell observed that an effective *metric* to measure likeness and differences between sensory stimuli is essential for the advancement of any sensory science [3]. Even in artificial intelligence, one of the fundamental questions we address is “what makes sensory inputs (images or speech) seem alike or different”. While numerous studies are conducted to identify standard metrics to measure the human-perceived (dis)similarity between images [4–6], speech [7, 8], and tactile signals [9], olfaction and taste (also called chemical senses) lack such metrics to quantify, characterize, or distinguish sensory stimuli [10]. Our study makes an effort in this direction to quantify and characterize perceptual dissimilarity between odorants by employing machine learning techniques.

One of the critical steps toward developing perceptual metrics is to learn the perceptual features of input stimuli. In the audiovisual domain, wavelength and frequency enable the characterization of perceptual features such as color and pitch. In contrast, learning olfactory perceptual features is much more challenging. Several prior studies [11, 12] made an effort to derive a standard set of olfactory features that contributes to perception. However, their findings could not reach any reliable consensus due to an enormously large set of potential olfactory primaries that could be derived from hundreds of olfactory receptors.

Another approach to estimating the dissimilarity between odorants involves employing quantitative structure-odor relationship models (QSOR) [13]. The core idea of QSOR models is to identify *what structural features evoke a specific odor perception, say, “musky” or “fruity”*. Once an accurate mapping between structural features and odor percepts is learned, one can efficiently estimate the perceptual dissimilarity between odorants. QSOR models can potentially be used for other applications as well, such as synthesizing novel flavors/odors and drug discovery. However, despite the decade-long effort, a reliable structure-odor relationship has yet to be established. Numerous earlier attempts in this direction could see only limited success due to a) lack of chemoinformatic and computational tools to extract meaningful features of molecules and b) a complex relationship between odorant structure and percepts which is hard to model accurately. Furthermore, QSOR modeling necessitates discrete verbal descriptors for various odor impressions, which are challenging to gather and require domain-expert knowledge.

The emergence of modern chemoinformatic and computational tools such as Dragon [14] and ChemoPy [15] enabled easy access to thousands of physicochemical features. With remarkably powerful modeling tools such as deep learning, QSOR modeling has made notable progress. For instance, Benjamin *et al*. [16] built a QSOR model to learn perceptual features using Dragon [14] features and graph neural networks. Similarly, Kowalewski *et al*. [17] train the random forest [18] on the Dragon [14] features for odorant classification task. Although QSOR models can be used to estimate perceptual dissimilarity between odorants, they suffer from a few major disadvantages. First, they rely on *discrete* perceptual descriptors (“woody” or “musky”), which lack *granularity* to adequately capture the *continuity* of perceptual space. Moreover, such verbal profiling of odorants requires expert annotation, which is often expensive. They are also likely to be culturally and regionally biased due to the dependency on language-based verbal attributes. Arctander’s study [19] indicates that olfaction labels are usually obtained from certain domain specialists, such as perfumers or wine tasters, which does not represent the general perception of the population as a whole. Second, we emphasize the distinctiveness of our study in comparison to QSOR research [13, 16, 17, 19] that focuses on learning a *discrete* embedding space for odor classification. In contrast, our study is designed to learn a continuous perceptual space that can distinguish between two odorants even from the same class, based on their degree of differentiation. By adopting this approach, we are able to offer a more nuanced and comprehensive understanding of odor perception.

**Fig 1:**
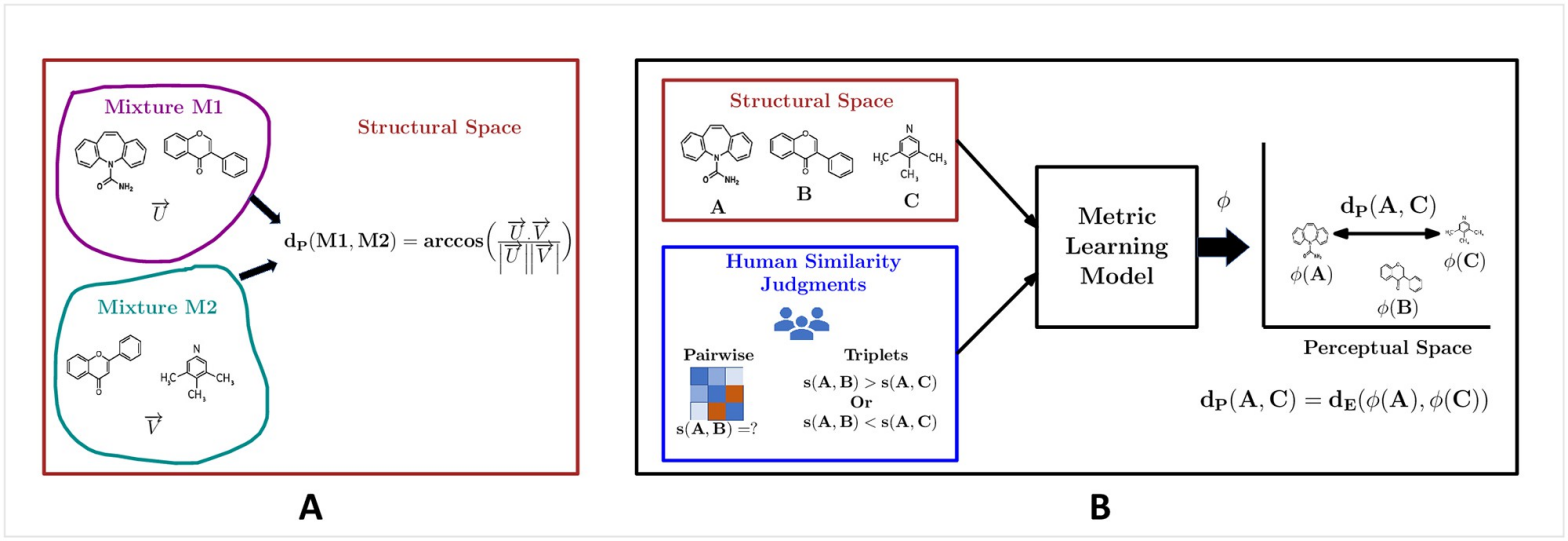
Comparison of Kobi’s approach *et al*. [20] with our perceptual metric learning approach (A) Kobi *et al*.: determine perceptual dissimilarity as a function of structural features. (B) Our model: combines structural features with perceptual judgments to learn a embedding function *ϕ*, which reflects the human-perceived odorant dissimilarity.

Our work is closely related to the papers by Kobi *et al*. and Ravia *et al*. [20, 21], both of which propose to use the cosine distance function on the structural features of odorants to estimate perceptual dissimilarity. While Kobi *et al*. [20] approximate the perceptual dissimilarity (*d*_*P*_) between mono-molecular odorants, Ravia *et al*. [21] extend the same study on the mixture of odorants by combining multiple mono-molecular odorants. They demonstrate the effectiveness of the angular distance between structural features, which describe the physicochemical properties of odorants, in representing perceptual dissimilarity between them, as shown in Fig 1A. While the association between structural and perceptual attributes is plausible, defining perceptual dissimilarity solely as a function of structural features seems improbable, especially considering that odor perception may be subjective. Both studies [20, 21] make an assumption on the correlation between structure and perception—similarity in structural space leads to the similarity in perceptual space. However, this is not necessarily true, as structural similarity may not ensure perceptual similarity. In fact, even a minimal difference in a molecule’s structure can significantly change its odor, the phenomenon is commonly known as an activity cliff [22] in the olfaction literature.

Our study avoids such assumptions and proposes three different machine learning based approaches to perceptual metric learning (PMeL). In contrast to Kobi *et al*. and Ravia *et al*. [20, 21] works, our framework combines both structural features and perceptual similarity judgments to estimate the human-perceived dissimilarity between odorants, as shown in Fig 1B. All three techniques attempt to learn an embedding function *ϕ* on molecular feature space using supervision derived from non-expert perceptual similarity feedback. Subsequently, a simple Euclidean distance approximates the perceptual dissimilarity between the odorants in the learned embedding space, as shown in Fig 1B. Our work is motivated by the need to avoid reliance on discrete verbal profiling of odorants, which is much more challenging to obtain, especially in the olfactory domain. Avoiding discrete verbal profiling of odorants, our method instead captures the notion of perceptual dissimilarity from triplet relative similarity comparisons obtained from non-experts: “does molecule A smell more similar to molecule B, or molecule C?” Such triplet comparisons pose several significant advantages—they are more consistent across cross-cultural users, do not require domain experts for annotation, reduce cognitive load for labelers, moderate the odds of calibration errors, and are easier to obtain even from non-expert users [23, 24]. Our main contributions are as follows.

1. We propose to combine molecular structural features with the user’s perceptual similarity feedback to learn the olfactory perceptual space.
2. Our method avoids the need for discrete perceptual descriptors and derives supervision from relative comparisons, which are effective in modeling the continuous perception space and do not require expert annotation. The concept of learning a perceptual embedding space through relative similarity comparisons has been explored in the fields of computer vision and speech processing. However, our study is the first to investigate its applicability for modeling the olfactory perceptual space.
3. We present a comparative study of various distance measures, including non-parametric (multidimensional scaling [25]), linear-parametric (Mahalanobis), and non-linear-parametric (Deep metric), to quantify human-perceived dissimilarity between odorants. We then evaluate each metric against other standard baselines for several dissimilarity assessment tasks and present important insights into their effectiveness in diverse experimental scenarios.
4. At last, we examine how the similarity predictions by our model align with similarity feedback provided by domain experts. Insights in that direction offer a new perspective on ways to reduce reliance on expert-annotated data.

## Methods

This section describes three approaches to perceptual metrics learning. First, we propose to use a classical non-parametric approach, multidimensional scaling (MDS [25]), which has been extensively used in psychometric studies [26, 27] to estimate the human-perceived dissimilarity. However, its utility, at present, seems to have been largely unexplored in olfactory research. Further, to handle out-of-sample extensions, we propose two parametric approaches to perceptual metric learning. In all three methods, we learn a representation of input odorants that effectively mimic human perceptual similarity judgments on odorants.

### Multi-dimensional scaling (MDS)

Given a set of *N* molecules $X={\left\{x_{i}\right\}}_{1}^{N}\in R^{d}$ represented by *d* dimensional features and pairwise dissimilarity matrix *D*, where each entry *d*_*ij*_ indicates the user-perceived dissimilarity between odorant *i* and *j*, our goal is to learn a representation $\hat{X}={\left\{\hat{x_{i}}\right\}}_{1}^{N}\in R^{\hat{d}}$ of the input odorants in a way that if users perceive that odorant *i* smells more like *j* than *k*, then the Euclidean distance *d*_*E*_ should satisfy $d_{E}\left(\hat{x_{i}},\hat{x_{j}}\right)<d_{E}\left(\hat{x_{i}},\hat{x_{k}}\right)$. The above formulation tries to learn a spatial representation ($\hat{X}$) of input odorants in a low-dimensional space so that inter-sample Euclidean distances conform with the user-specified dissimilarities between odorants. Due to inherent subjectivity in human perceptual judgments, it is challenging to learn an input representation that aligns with the similarity judgments of all users. Therefore, instead of preserving *absolute* similarity values, we aim to satisfy only *relative* similarity orderings, which are easier to model and less prone to subjectivity.

The method involves iterative steps to minimize the stress function representing the difference between observed *d*_*ij*_ and learned dissimilarity $d_{E}\left(\hat{x_{i}},\hat{x_{j}}\right)$, using gradient descent [28]. The learned embedding $\hat{X}$ is rearranged at each iteration to maximize the ordination fit to the user’s similarity judgments [29]. This, in turn, implies the minimization of stress function *S* expressed as Eq 1.

$$\begin{array}{c} \begin{array}{c} S=\sqrt{\frac{\sum {\left(d_{ij}-d_{E}\left(\hat{x_{i}},\hat{x_{j}}\right)\right)}^{2}}{\sum d_{ij}^{2}}}\;\text{where}\;d_{ij}=\text{perceived}\;\text{dissimilarity}\\ \;d_{E}\left(\hat{x_{i}},\hat{x_{j}}\right)=\text{estimated}\;\text{dissimilarity} \end{array} \end{array}$$ (1)

Here, the constraints are ordinal. More precisely, whenever *d*_*ij*_ < *d*_*ik*_, it implies $d_{E}\left(\hat{x_{i}},\hat{x_{j}}\right)<d_{E}\left(\hat{x_{i}},\hat{x_{k}}\right)$. Once the embedding, $\hat{X}$, is learned, the Euclidean distance between the learned perceptual features, $d_{E}\left(\hat{x_{i}},\hat{x_{j}}\right)$, represents the perceptual distance *d*_*P*_(*x*_*i*_, *x*_*j*_). Although MDS is effective in modeling perceptual dissimilarity, it is limited in predicting dissimilarity between new unseen samples. Moreover, it fails to capture non-linear structure in the data. This motivates our study to explore parametric approaches that enable easy extension to unseen test samples.

### Mahalanobis metric

Given input odorants $X={\left\{x_{i}\right\}}_{1}^{N}\in R^{d}$ and user-specified perceptual similarity judgments, our objective is to learn a linear distance function $W:R^{d}\to R^{\hat{d}}$ such that *d*_*E*_(*W*^*T*^*x*_*i*_, *W*^*T*^*x*_*j*_) < *d*_*E*_(*W*^*T*^*x*_*i*_, *W*^*T*^*x*_*k*_) if the odor impression of molecule *x*_*i*_ is more similar to *x*_*j*_ than *x*_*k*_. In other words, the perceptual distance, as described in Eq 2, can be viewed as the squared Euclidean distance on the linearly transformed molecular features *W*^*T*^*x*.

$$\begin{array}{c} \begin{array}{cc} d_{W}\left(x_{i},x_{j}\right) & =\|Wx_{i}-Wx_{j}{\|}_{2}^{2}={\left(x_{i}-x_{j}\right)}^{T}W^{T}W\left(x_{i}-x_{j}\right)\\ d_{M}\left(x_{i},x_{j}\right) & ={\left(x_{i}-x_{j}\right)}^{T}M\left(x_{i}-x_{j}\right)\;\text{where}\;M=W^{T}W \end{array} \end{array}$$ (2)

The resultant distance is parameterized by a positive definite matrix *M* = *W*^*T*^*W*. Note that our framework saves the effort and cost of obtaining numerical estimates of similarity between molecules as ground truth values. Instead, we consider triplet similarity constraints (*i*, *j*, *k*) ∈ *C* obtained from users, which indicates the smell perception of the odorant *i* is more similar to the odorant *j* than the odorant *k*. For given perceptual feedback as relative similarity constraints *C* and molecular structural features *X*, we develop an optimization framework that maximizes the distance margin, $d_{M}^{2}\left(x_{i},x_{k}\right)-d_{M}^{2}\left(x_{i},x_{j}\right)$, between dissimilar (*x*_*i*_, *x*_*k*_) and similar odorant pairs (*x*_*i*_, *x*_*j*_) for as many triplets (*i*, *j*, *k*) as possible. We use the gradient descent technique for optimization. Unlike the Euclidean distance, which assigns equal weights to all input dimensions, the Mahalanobis metric takes the correlation between input dimensions into account in the computation of distances. This enables it to effectively capture complex data semantics, as demonstrated in our experimental results. However, if the data is highly complex and non-linear, as in the case of human perception, then the Mahalanobis distance may not be powerful enough to effectively represent the odor perceptual dissimilarity. Our experimental results in the next section reflect this intuition. As a result, we employ a deep neural network for learning an appropriate embedding function that maps input to non-linear feature space, preserving perceptual dissimilarity between odorants.

### Deep metric

**Fig 2:**
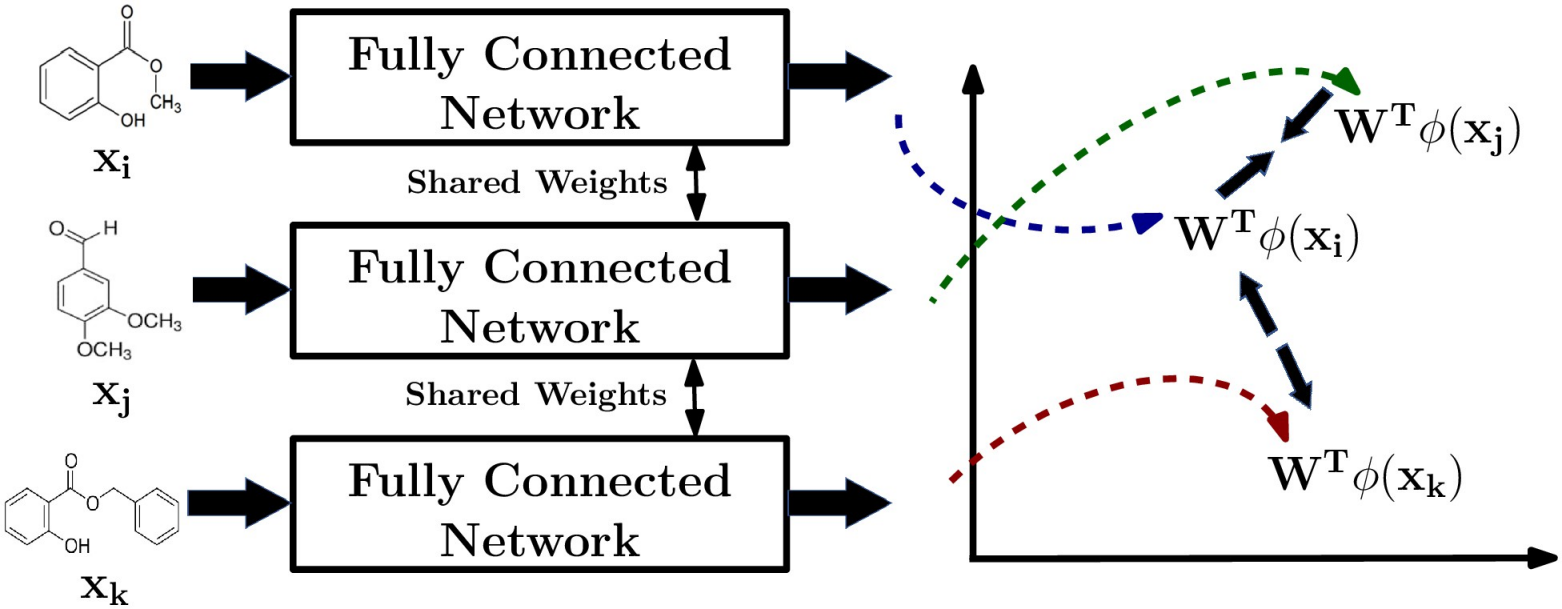
An illustration showing how a triplet network works to bring perceptually similar odorants closer together and distinct ones further apart in the learned embedding space *ϕ*.

Similar to the Mahalanobis metric, the Deep metric learns an explicit parameterized dissimilarity function in the input feature space using the supervision derived from the user’s perceptual similarity feedback captured as triplet constraints. Unlike non-parameteric methods, deep metric allows dissimilarity computation for unseen test samples without retraining the model from scratch. To effectively model the complexity of perceptual data, we learn a non-linear embedding function $\phi :R^{d}\to R^{\hat{d}}$ followed by a linear distance metric *M* in the transformed feature space. Our framework as shown in Fig 2 provides an end-to-end training framework by combining kernel *ϕ*(*x*) and metric *M* learning together. Our neural network architecture (Fig 2) is inspired by prior works [6, 9], which have been proven effective for perceptual similarity learning for images and haptic signals. Our architecture is similat to siamese network [30], consisting of three replicas of the network with shared weights, each composed of three fully-connected layers with ReLU nonlinearities except the last layer, which has linear activation and zero bias. The choice of our network architecture provides an end-to-end training framework, which efficiently combines non-linear embedding function learning *ϕ* using a compositional network up to the penultimate hidden layer and metric learning *M* using the last linear layer at the end. Overall the entire framework can still be viewed as learning the Mahalanobis distance on the non-linearly transformed features *ϕ*(*x*) obtained from the second-last layer of the network: $d_{P}\left(x_{i},x_{j}\right)=d_{M}^{2}\left(\phi \left(x_{i}\right),\phi \left(x_{j}\right)\right)$.

As shown in Fig 2, three molecules of a triplet (*i*, *j*, *k*) ∈ *C* are fed to three separate branches of the network, and the network is trained using the exponential triplet loss function *L*(*x*_*i*_, *x*_*j*_, *x*_*k*_) as described in Eq 3. Our loss function is optimized by bringing similar odorants (*x*_*i*_, *x*_*j*_) closer and dissimilar ones (*x*_*i*_, *x*_*k*_) apart.

$$\begin{array}{c} \begin{array}{c} L_{\phi ,M}\left(x_{i},x_{j},x_{k}\right)=\sum_{\left(x_{i},x_{j},x_{k}\right)}e^{-(d_{M}^{2}\left(\phi \left(x_{i}\right),\phi \left(x_{k}\right)\right)-d_{M}^{2}\left(\phi \left(x_{i}\right),\phi \left(x_{j}\right)\right)}\\ \text{where}\;d_{M}^{2}\left(\phi \left(x_{i}\right),\phi \left(x_{j}\right)\right)={\left(\phi \left(x_{i}\right)-\phi \left(x_{j}\right)\right)}^{T}M\left(\phi \left(x_{i}\right)-\phi \left(x_{j}\right)\right) \end{array} \end{array}$$ (3)

#### Hyperparameters

Our model is trained with Adam optimizer [31] and a learning rate of 0.001 for 1000 epochs. The hyperparameters, including the learning rate (0.001), batch size (200), and number of epochs (1000), are empirically tuned. We also experimented with triplet margin loss and found exponential triplet loss performing better in our case. Both Mahalanobis and Deep distance function generalize well on new unseen data and do not require manual feature selection. Next, we introduce the dataset used in our study and the evaluation setup before discussing the effectiveness of each approach in the results section.

#### Dataset

**Fig 3:**
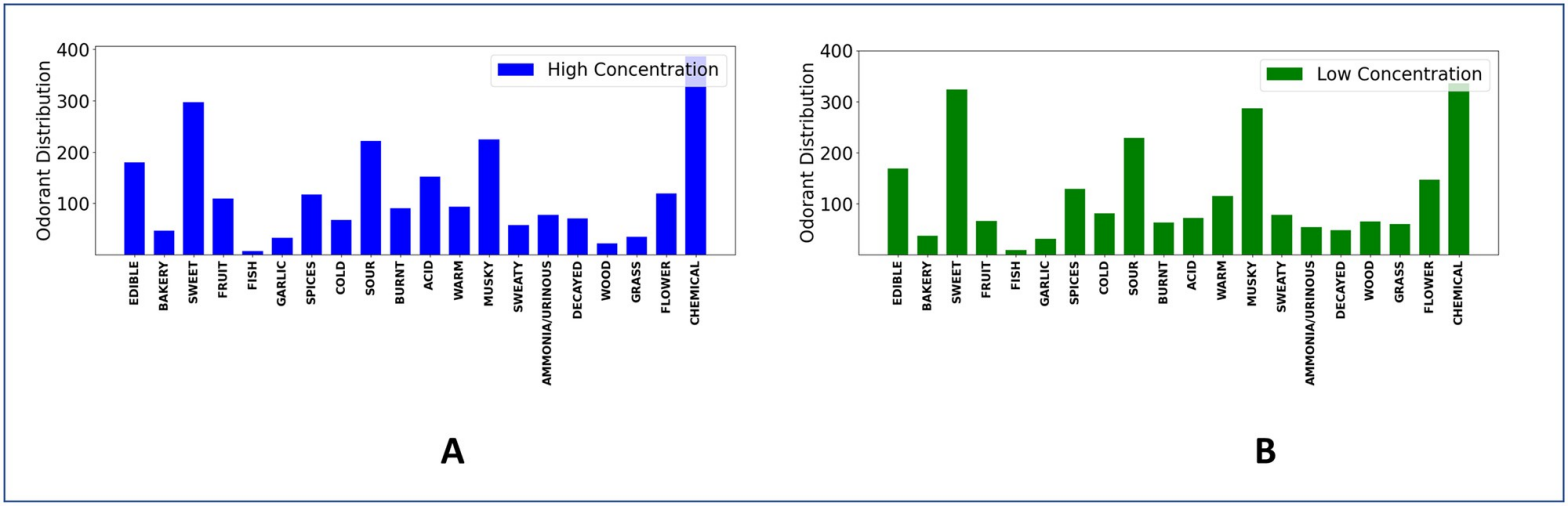
Distribution of perceptual descriptors of odorants at high (blue) and low (green) concentrations. Few descriptors, such as “sweet”, “chemical”, and “musky” are more often used to describe odor perception than other descriptors.

We use publicly available dataset by Keller *et al*. [32] sourced from the DREAM Olfaction Prediction Challenge [33]. The dataset consists of 480 structurally diverse molecules at two different concentrations. For each molecule, perceptual similarity ratings are obtained from 55 subjects of diverse ethnicity, age, and gender. The perceptual ratings are collected on 20 odor descriptors—“edible”, “bakery”, “sweet”, “fruit”, “fish”, “garlic”, “spices”, “cold”, “sour”, “burnt”, “acid”, “warm”, “musky”, “sweaty”, “ammonia/urinous”, “decayed”, “wood”, “grass”, “flower”, and “chemical” on an analog scale from 0–100, with 0 and 100 indicating *absence* and *highest prominence* of an odor descriptor, respectively. Unlike the binary dataset that indicates just the presence or absence of an odor percept, the Keller dataset presents richer information by providing a degree of presence of a particular odor percept on an analog scale, which makes it better suited for capturing the granularity of the olfactory perceptual space. The final ground truth rating is computed by taking an average of all 55 subjects’ ratings across all 20 descriptors. Hence each molecule *i* is represented by a 20 dimensional continuous descriptor vector ($\vec{y_{i}}\in R^{20}$). Fig 3 shows the descriptors distribution for all odorants at two different concentrations. As we can see, the dataset is skewed, with “chemical”, “sweet”, “musky”, “edible”, and “sour” more often used than others at both high and low concentrations.

#### Relative similarity comparisons

Note that our learning framework does not require discrete odor descriptors to learn the perceptual metrics for odorants. In fact, the motivation of our proposed method is to avoid discrete verbal descriptors for several shortcomings such as high subjectivity and ambiguity. The Keller *et al*. dataset does not provide triplet relative similarity between odorants. It instead captures pairwise dissimilarity between odorants from users. Therefore, first, we generate a set of triplets comparison *C* using a given dataset. We compute the pairwise similarity between all pairs of odorants (*i*, *j*), using the cosine function: $d_{ij}=\frac{\vec{y_{i}}\cdot \vec{y_{j}}}{\|\vec{y_{i}}\left\|\right\|\vec{y_{j}}\|}$. Here $\vec{y_{i}}\in R^{20}$ is the perceptual descriptor vector of the odorant *i*. Using the pairwise dissimilarities for all odorant pairs, we construct an exhaustive set of triplet constraints *C* = (*i*, *j*, *k*)|*d*_*ij*_ < *d*_*ik*_, indicating user-perceived dissimilarity of molecule-pair (*i*, *j*) is less than the molecule-pair (*i*, *k*).

#### Evaluation setup

We consider several state-of-the-art structural features *X* as our baseline—Keller *et al*. feature (used in the paper [32]), Mordred [12], and Dragon [14] features. The dimension of the Keller features is 379. To make the comparison fair, we reduce the dimension of the Mordred and Dragon features to 379 using principal component analysis (PCA) and singular value decomposition (SVD). Our models are trained on 379 dimensional structural features *X* with perceptual supervision derived from user similarity feedback encoded as pairwise dissimilarity matrix *D* or triplet comparisons *C*. We follow standard training practice; we create ten splits of the dataset, consisting of training, validation, and test sets, and evaluate each model on the test set. We measure the model performance on ordinal similarity ranking by the *triplet generalization accuracy (TGA)*, which indicates the percentage of test triplets (ground-truth) whose triplet similarity orders, given by users, match with the similarity orderings predicted by our learned model. For pairwise dissimilarity, we assess the performance of all three metrics, MDS, Mahalanobis, and Deep metrics, using the learned measures $d_{E}^{2}\left(\hat{x_{i}},\hat{x_{j}}\right)$, $d_{M}^{2}\left(x_{i},x_{j}\right)$, and $d_{M}^{2}\left(\phi \left(x_{i}\right),\phi \left(x_{j}\right)\right)$, respectively. Further, we demonstrate the qualitative performance in mimicking perceptual dissimilarity between odorants using confusion matrices.

## Results and discussion

This section presents a set of experiments that we have carried out to evaluate the performance of the three metrics on various similarity assessment tasks defined on odorants at two different concentrations. Due to space constraints, we report some experimental results in the Supporting Information. We first evaluate the effectiveness of the Multidimensional Scaling (MDS) approach in representing the human-perceived dissimilarity between odorants. Subsequently, we assess the Mahalanobis and Deep metrics in representing human perception by answering the following questions: **Q1**: How effective are our learned metrics in emulating ordinal (relative) similarity provided by users **Q2**: Can the learned metrics also preserve pairwise perceptual distances? **Q3**: How effective is our framework in the retrieval task? Subsequently, we perform qualitative analysis to highlight some key observations of our study. For instance, **Q4**: How does the concentration of odorants affect smell perception? **Q5**: How well does similarity predicted by the perceptual models trained on non-expert similarity feedback align with expert similarity assessments? In addition, we investigate the performance of different physicochemical features in emulating human perception.

### Performance on ordinal similarity ranking (Q1)

**Fig 4:**
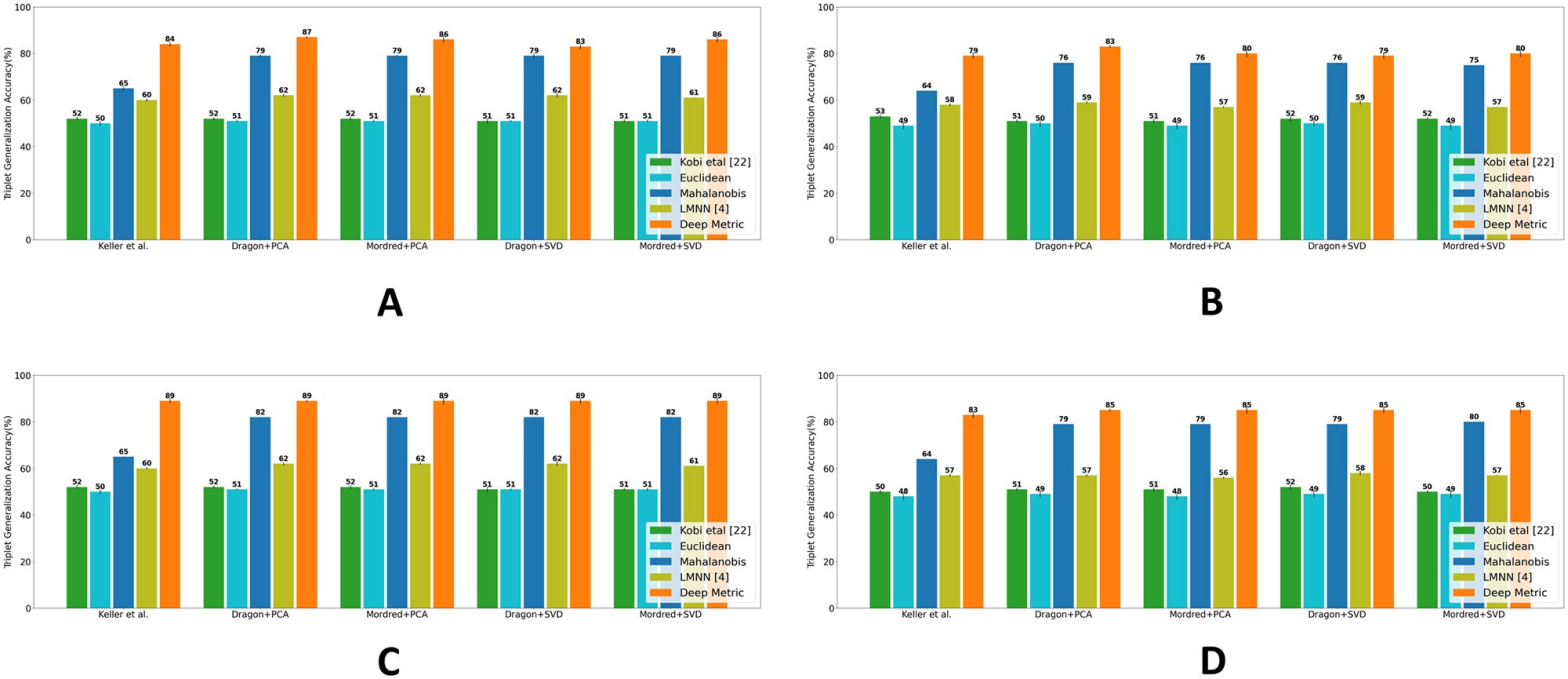
Performance comparison on relative similarity comparison task. For all features, the model performance is measured by the mean triplet generalization accuracy over ten splits of data. **A**: High concentration w/o “pleasant” descriptor **B**: Low concentration w/o “pleasant” descriptor **C**: High concentration with “pleasant” descriptor **D**: Low concentration with “pleasant” descriptor. Our study indicates the importance of the “pleasant” descriptor—its inclusion in the ground-truth similarity computation improves the performance of metrics at both concentrations.

**Fig 5:**
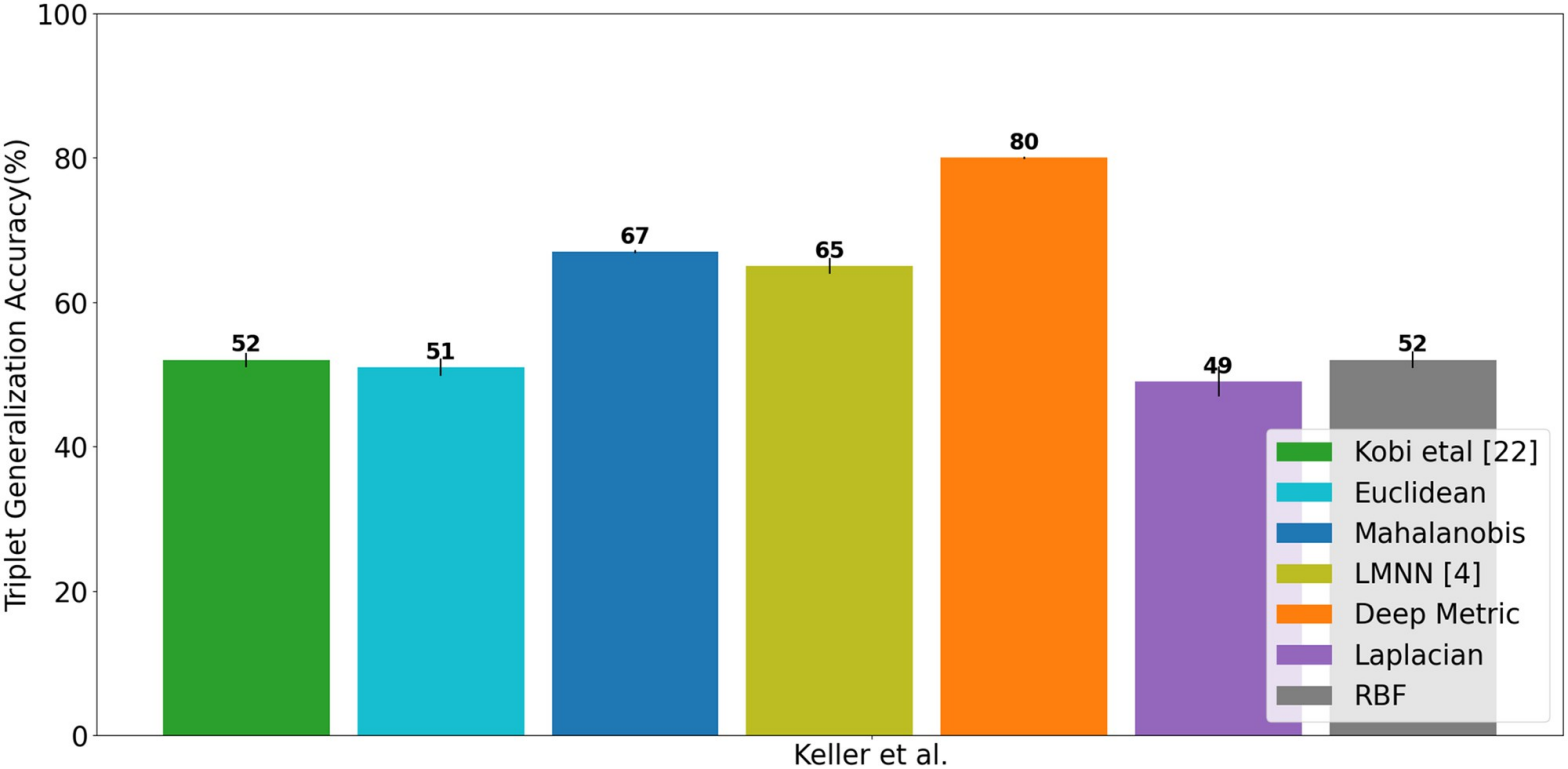
Triplet generalization accuracy of different metrics on Goodscent dataset.

We start by investigating the effectiveness of MDS on the ordinal similarity ranking task. We observe that MDS is quite effective in representing perceptual similarity between odorants, with a TGA accuracy of 89.8 ± 0.007% at high and 85.2 ± 0.007% at low concentrations. On the other hand, other non-parametric measures such as spectral embedding and t-SNE [34] performed sub-optimally, with an accuracy of only 48 ± 0.008% and 57 ± 0.005% for low concentration odorants, and 51 ± 0.006% and 58 ± 0.002% for high concentration odorants, respectively. Next, we compare the performance of Mahalanobis and Deep metrics against other baseline metrics and prior approaches in the literature, including the largest margin nearest neighbor (LMNN), Euclidean distance, and cosine distance used by Kobi *et al*. and Ravia *et al*. [20, 21]. We train the LMNN [4], Mahalanobis and Deep metrics on ten splits of training triplets, resulting in 10 perceptual models, and report the mean accuracy and variance of each metric over ten runs. As shown in Fig 4, our learned metrics, both Mahalanobis and Deep metrics, perform consistently well across all structural feature types, with Dragon features reduced by PCA performing slightly better than other choices of features and data reduction techniques. We validated the robustness of our method by performing the same experiment on another dataset, Goodscent. In line with the Keller *et al*. [32] dataset, we initially transformed the descriptor ratings into triplet similarity ordering using Jaccard similarity and proceeded to utilize the same methodology for model training. To provide further clarification, it should be noted that unlike the Keller dataset, we do not have descriptor annotation from non-expert users for two concentrations. Moreover, the Goodscent annotation is coarse, representing only the presence or absence of a particular smell impression. As a result, the representation for the molecules is binary, in contrast to the more fine-grained, continuous descriptor vectors used in the Keller dataset. As shown in Fig 5, we observed a similar performance trend, albeit with slightly degraded accuracy for all metrics. This is expected since the ground-truth annotation is coarse, and that leads to sub-optimal modeling of the continuous perceptual space.

Several studies [35], including Keller *et al*. [32], consider the odor descriptor “pleasant/unpleasant” as the most prominent dimension in odor perception. Therefore, we were curious to check how the hedonic descriptor “pleasant” contributes to modeling perceptual similarity. We generate a new set of triplets with 21 odor descriptors ($\vec{y}\in R^{21}$) including the descriptor “pleasant” and train the Mahalanobis and Deep model from scratch. As shown in Fig 4C and 4D, we observe a gain, up to 6%, in the model performance with the inclusion of the “pleasant” descriptor. Our study supports the previous finding and reaffirms that the “pleasantness” attribute is crucial and improves the perceptual distinguishability of odorants.

#### Discussion

As shown in Figs 4 and 5, the deep metric significantly outperforms all other metrics, followed by Mahalanobis and LMNN for all features at both high and low concentrations. This result demonstrates the neural network model’s ability to model non-linear and complex relationships in the data. Our study also revealed some interesting observations that align with our intuition. Specifically, we found that the Mahalanobis metric performs better than the Euclidean and cosine functions because it takes into account the correlation between different feature dimensions, whereas Euclidean and cosine treat all components as independent and identical distributed. Additionally, we observed that the deep metric outperforms Mahalanobis and LMNN due to its ability to capture non-linear and complex semantics in the data.

We also observe the performance of the learned metrics for high-concentration odorants is slightly better than that for the lower concentration. We believe that the user’s perceptual rating is less ambiguous at a high concentration, leading to superior metrics performance. Note that MDS performs on par with the Deep metric. In general, MDS is effective in capturing implicit hidden concepts that humans use while making similarity judgments. For instance, users may group even chemically distinct odorants based on their familiarity. Such vague perceptual concepts are hard to capture by structural features. MDS gains advantage in modeling such concepts as the method is not restrained by structural features. MDS enables learning perceptual features in an abstract space that abides maximally with perceptual constraints. In contrast, parametric approaches are tied by learning an embedding function in the input molecular feature space. We observe a slightly superior performance of MDS even in the pairwise distinguishability task described in the next subsection. In summary, we achieve comparable performance of MDS and Deep metric, but unlike MDS, Deep metric has generalization ability on new samples. Due to superior performance and better generalization ability, all the results shown subsequently are on the Dragon features reduced by PCA and triplets derived from 21 perceptual descriptors (including “pleasant”), unless otherwise stated.

### Performance on pairwise similarity (Q2)

**Fig 6:**
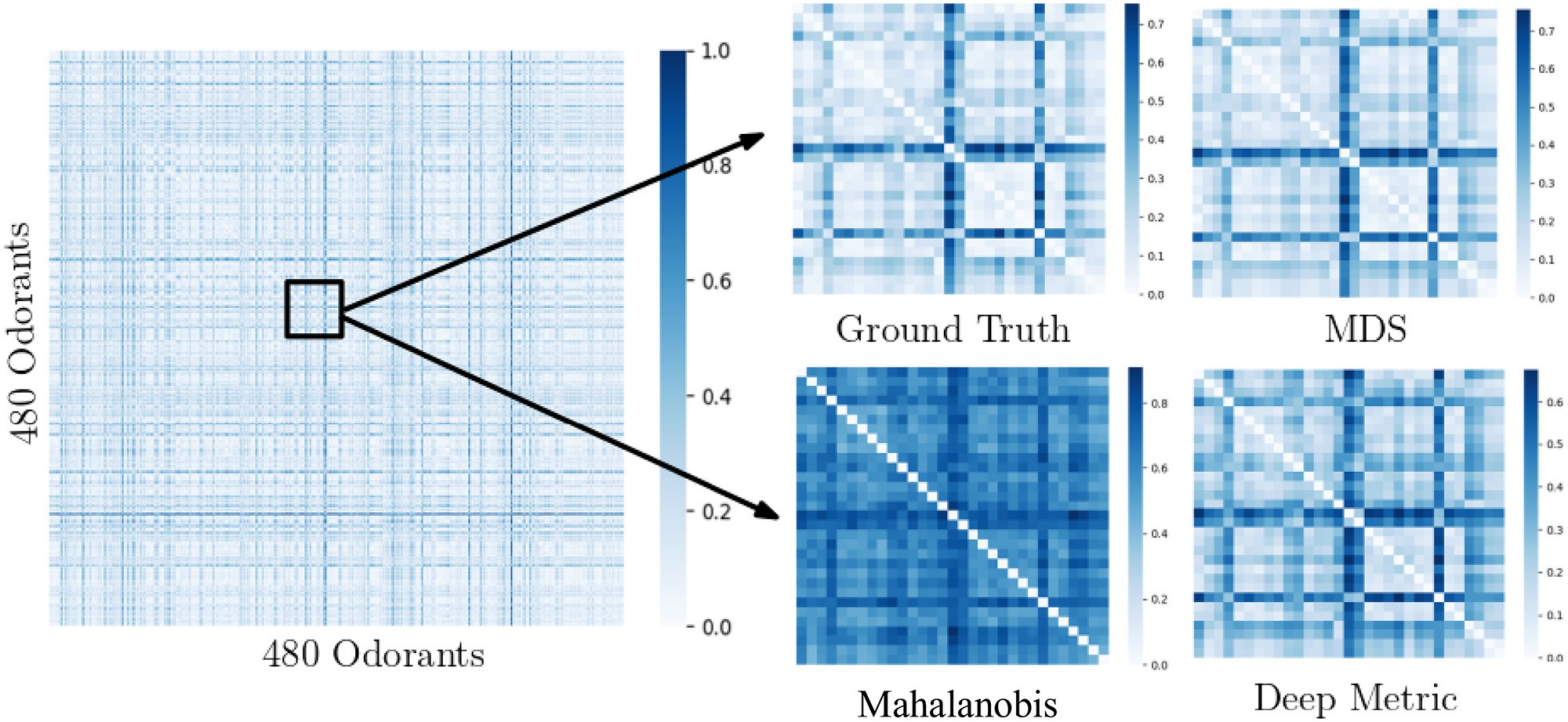
Performance of different perceptual metrics on pairwise distinguishability task. The left-most confusion matrix shows the ground truth similarity generated using the users’ study data conducted by Keller *et al*. [32]. The matrix is normalized between 0 and 1, with 0 and 1 indicating the most similar and dissimilar pairs, respectively. To improve visualization, we enhance a particular region to show the pairwise distinguishability of different metrics.

Now, we evaluate how effective different dissimilarity measures are in preserving pairwise distances in the learned embedding space. An accurate representation of perceptual pairwise distance can enable several applications, which require identifying distinguishable from indistinguishable odorant pairs, such as novel perfume or flavor synthesis. As mentioned earlier, the ground truth similarity is computed using cosine similarity on average descriptor ratings on 480 odorants by Keller *et al*. [32]. Fig 6 shows the ground-truth (GT) pairwise distances for all 480 odorants (left) at high concentration (a similar result for low concentration is shown in the Supporting Information). For better visualization, we randomly select a region in the ground truth confusion matrix and enhance it to compare the performance of different metrics. Each entry in the confusion matrix denotes the normalized average pairwise distance between corresponding odorants across multiple runs. As we see in Fig 6, the pairwise distances between features learned by MDS or Deep metric matches the ground-truth values quite well.

#### Discussion

While Deep metric and MDS quite effectively replicate the general trend in similarity values of ground truth, they do not precisely reproduce the exact numerical estimates of similarity. This aligns with our intuition as our framework optimizes for preserving relative similarity ordering of odorants. The model is trained on ordinal similarity orderings; hence, we expect better match in ordinal similarities instead of quantified similarities. Noting the superior performance of the Deep metric over the Mahalanobis metric in capturing pairwise and ordinal similarity learning of odorants, we further investigate the performance of the Deep metric on other defined tasks such as perceptual embedding and odorant retrieval.

### Perceptual embedding and retrieval task (Q3)

**Fig 7:**
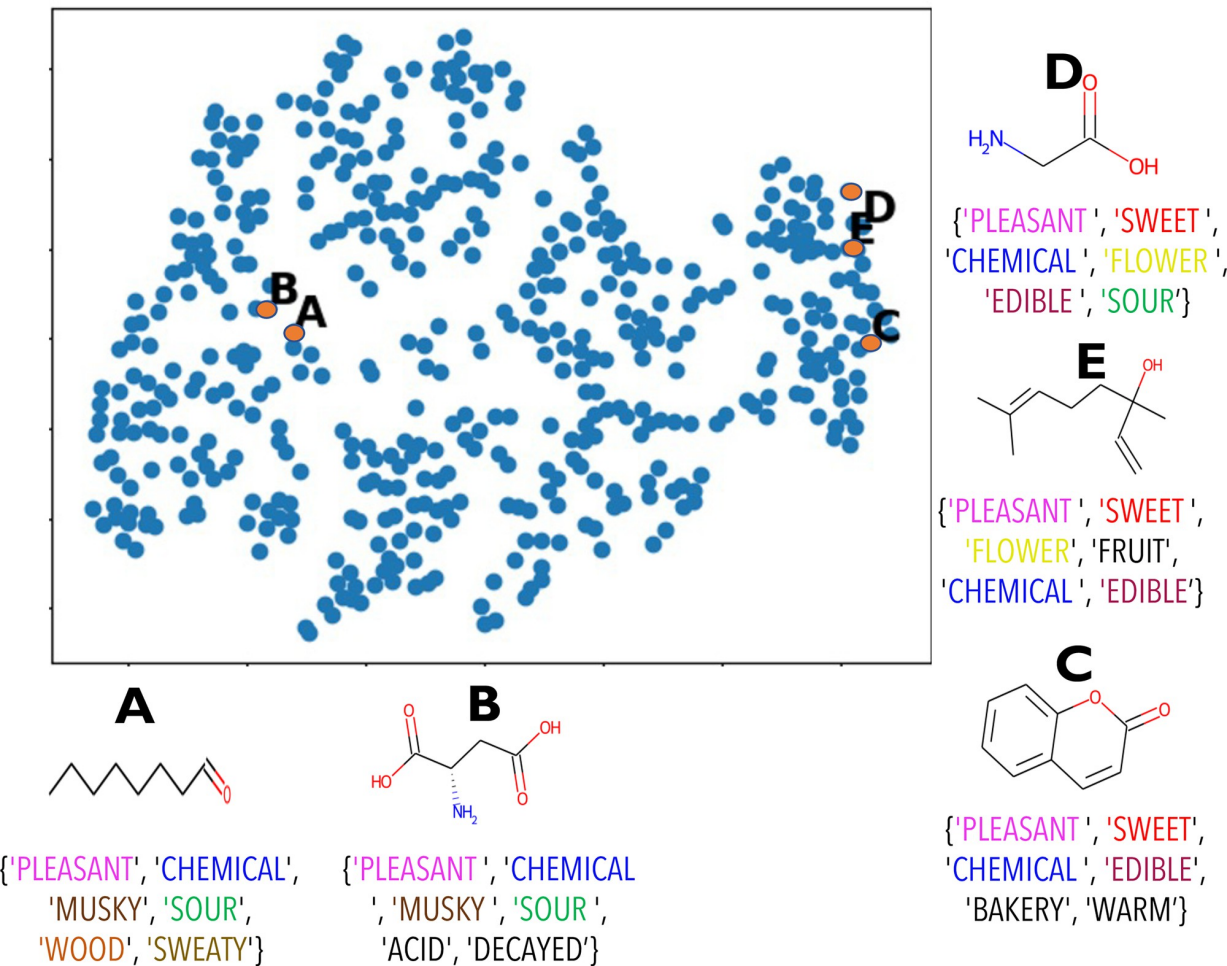
2D projection of molecules in the learned space using t-SNE. Few odorants are highlighted to show how the placement of molecules in the embedding space reflects the perceptual ratings gathered from users.

To evaluate the effectiveness of the learned perceptual embedding space as a whole, we visualize the representation of odorants learned by our Deep metric using the t-SNE algorithm [34]. The learned Deep metric outputs a 25 dimensional representation of odorants, which are embedded in 2*D* space via t-SNE mapping. Fig 7 shows all 480 high concentration odorants in the learned embedded space, where proximity between odorants indicates the degree of similarity in the smell perception. Few randomly selected odorants (shown in orange) and their six most-rated perceptual descriptors by non-expert users are explicitly highlighted for qualitative analysis. As we see in Fig 7, the overlap in the perceptual descriptors of nearby odorants is indeed greater than the distant odorants. A similar trend is also reflected in the learned perceptual space—*d*_*p*_(*A*, *B*) < *d*_*p*_(*A*, *D*), *d*_*p*_(*A*, *D*) ≈ *d*_*p*_(*A*, *E*), *d*_*p*_(*D*, *E*) < *d*_*p*_(*D*, *C*), etc. The descriptors “pleasant” and “chemical” are common for all selected odorants, as they have been the most frequently used by users to describe odorants.

**Fig 8:**
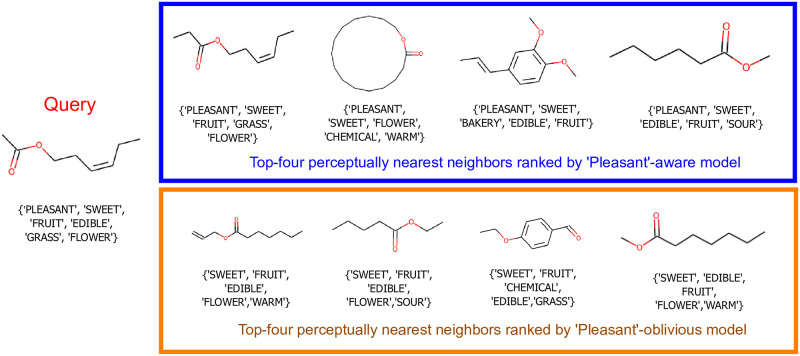
The top four perceptually similar odorants (in decreasing order from left to right) to query odorant retrieved by Deep metric. For the selected query, we show the results of two models trained w/ and w/o the “pleasant” descriptor. In both cases, our model could effectively retrieve perceptually similar odorants.

We further investigate how a model performs on the odorant retrieval task, which is more effective in evaluating the robustness of the learned model in identifying perceptually similar neighbors. We randomly select a query odorant and retrieve its nearby molecules in the learned embedding space. Fig 8 shows the top four closest neighbors of the query odorant in the learned embedding space. For each molecule, we show five perceptual descriptors rated by non-expert users. The top row lists the retrieved odorants in the embedding space when the “pleasant” descriptor is used in the training data. To check if the learned space is biased towards the hedonic descriptor “pleasant”, which is dominantly used by users, we trained a model by excluding it from the training data. As Fig 8 shows, both embeddings learned with or without the dominant descriptor are quite effective in retrieving perceptually similar odorants: this feature enables several applications, which require discovering a substitute for a given odorant.

#### Discussion

We observe that our learned embedding space shows a meaningful pattern. If we ignore dominantly used descriptors such as “pleasant” and “chemical”, molecules on the left side of the embedding space are malodorous, having “musky”, “sweaty”, and “acid” smell impression, whereas molecules on the right side are more pleasant-smelling, having odor impression of “sweet”, “flower”, “fruit”, etc. We also observe in Figs 7 and 8 perceptually similar odorants do not share the same functional groups. Our study further affirms that the perception of molecules involves much more complex mechanisms, and structural and/or physicochemical properties alone may be insufficient to fully characterize the olfactory perception. We also note that the correlation between some specific functional groups and their effect on odor impression is more frequently established than others. For instance, the presence of sulfurous or nitro group in a molecule often correlates to a pungent or unpleasant smell. However, other parameters, such as the number or relative position of functional groups or branching points, are equally crucial for how molecules are perceived as specific odors. Moreover, perceptual factors such as users’ familiarity with smell and subjective experience cannot be weighed less in modeling not just olfactory but any sensory perception. More results on retrieval with different functional groups are shown in the Supporting Information.

### Effect of concentration on smell perception (Q4)

**Fig 9:**
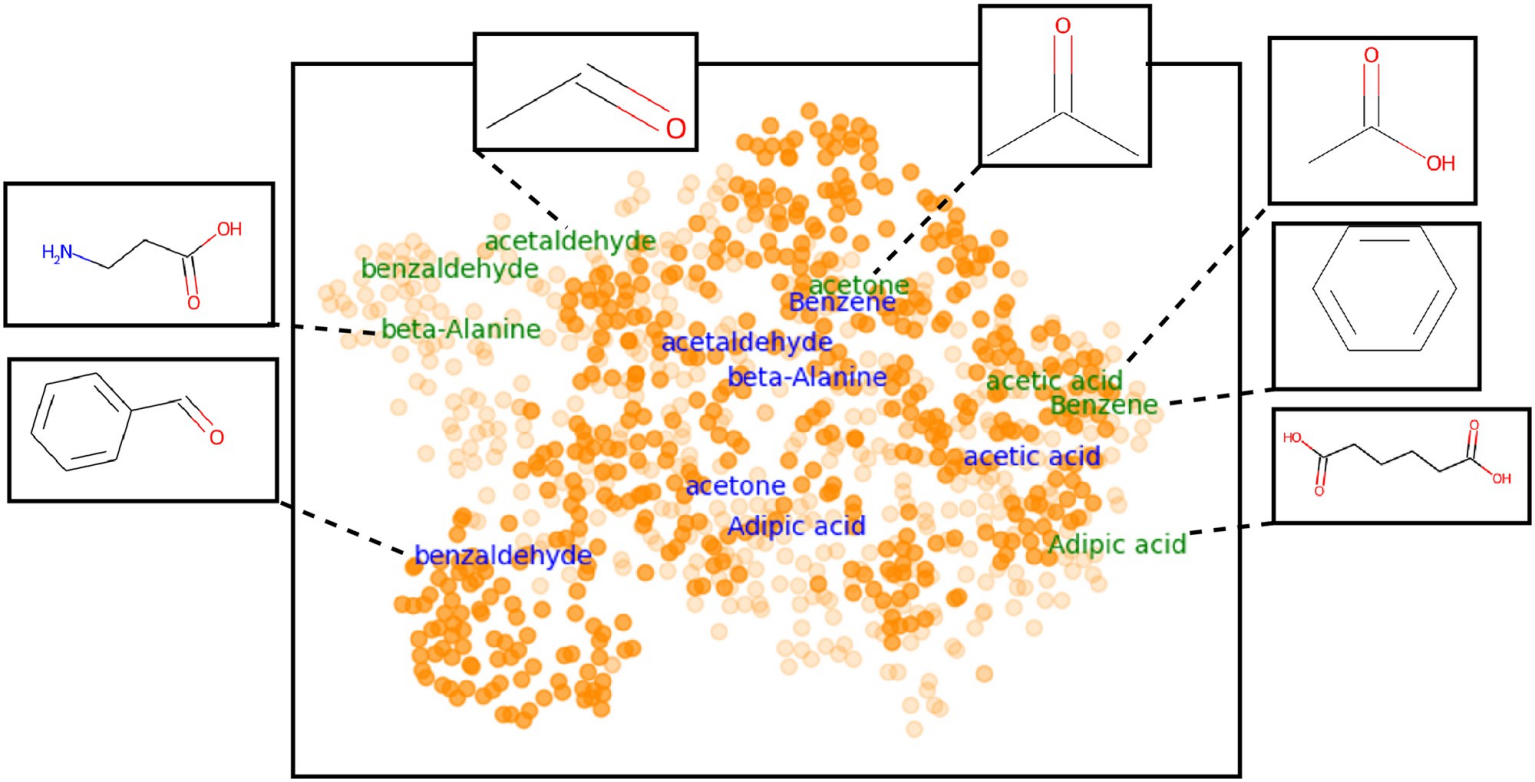
The learned features of 480 odorants at high (dark orange) and low (dim orange) concentrations are projected onto the 2D space using t-SNE mapping. Few randomly selected odorants at high concentration (blue text) and low concentration (green text) are highlighted to show the effect of concentration on the perceptual dissimilarity between molecules.

We now investigate how concentrations of molecules affect its smell perception. The Keller *et al*. [32] dataset contains odor descriptors from non-expert users for 480 molecules at two different concentrations. Based on the perceptibility of each odorant, non-expert users provide 21 dimensional perceptual ratings (including “pleasant” descriptor) at two different concentrations. We train two neural network models on triplet similarity constraints generated from perceptual ratings recorded for low- and high-concentration odorants. Using the t-SNE mapping, we project the 25 dimensional learned features into the 2*D* space. High- and low-concentration molecules are shown in dark and dim orange, respectively. It is interesting to note that the same molecule at two different concentrations sometimes is more dissimilar than the different molecules. For instance, in both ground-truth perceptual data and in the learned space, the smell perception of low-concentrated acetaldehyde is more similar to benzaldehyde (shown by green text) than high-concentrated acetaldehyde, shown in blue text in Fig 9. Intuitively, concentration affects smell perception: even an odorant with a strong smell can become odorless at high dilution. Moreover, some chemical properties, such as density and acidity, may change with concentration. Our results align with intuition and validate that smell perception is much more complex and a function of several physical, chemical, and perceptual factors than just its structure.

### Comparison of the learned model with expert feedback (Q5)

At last, we compare how closely similarity predicted by our learned model trained on non-expert users’ data conforms to expert similarity feedback. Unfortunately, olfaction lacks data that has direct similarity assessments on odorants from experts. We leverage the Goodscents data [36], which consists of thousands of odorants annotated by experts using a dictionary of discrete odor descriptors, to generate expert similarity assessments. The descriptor dictionary of the Goodscents is noisy, consisting of several equivalent descriptors, such as “fruit” and “fruity” and redundant connectors such as “with”, “like”, etc. We manually clean the data, leaving 255 odor descriptors for all molecules. Despite curation, a faithful comparison between Keller data used by our study [32] and Goodscents data [36] remains challenging for three reasons. First, the descriptor dictionary used in both datasets is very different. The Goodscents describe the odorants using 255 descriptors in contrast to just 20 descriptors used in the Keller dataset. The distribution of both is shown in the Supporting Information. Second, the annotation style of both datasets is different. Binary descriptor vectors describe the molecules in the Goodscents, whereas Keller data contains more fine-grained representation described by *continuous* descriptor vectors. Third, the non-experts annotated Keller data contains noise and a lot more subjectivity than the Goodscents data.

To address the first challenge, we consider only those descriptors that are common between the Keller and Goodscent data—16 descriptors are common in both and are mentioned in the Supporting Information. For uniformity in annotation style, we convert our data into binary descriptor vectors, discarding the scale ratings provided by users. Subsequently, we use the Jaccard distance as a common dissimilarity measure for both datasets. To moderate the noise and subjectivity in the Keller data, we discard infrequently used descriptors, i.e., used by less than 25% of the subjects. With the curated Keller data, we generate the triplets using the Jaccard distance and learn embedding of odorants using the proposed Deep metric. We validate the learned model using triplet generalization accuracy (TGA)—we check the percentage of test triplets (generated from the non-expert Keller data) on which our model’s prediction matches with the expert similarity assessments (generated from the Goodscents data). We observe that our model prediction matches fairly well with expert similarity assessments, with a TGA accuracy of 71%. Nevertheless, we further analyze the test triplets on which our model’s prediction differs from the expert data. A few examples of such triplets are shown in Table 1.

**Table 1:** Examples of test triplets on which the similarity ordering predicted by a model (trained on non-expert data) matches or differs from the expert similarity assessments derived from the Goodscents data.

S. No	Triplet of odorants {*A*, *B*, *C*}	Goodscents descriptors	Expert assessments	Predicted ordering
1	A—4-Isopropylbenzyl alcohol B—Ethyl cinnamate C—Benzyl disulfide	A—[‘spicy’] B—[‘spicy’, ‘sweet’, ‘fruit’] C—[‘burnt’]	*s*(*A*, *B*) > *s*(*A*, *C*)	*s*(*A*, *B*) > *s*(*A*, *C*)
2	A—Piperonal B—(1R)-(-)-Myrtenal C—4-Ethoxybenzaldehyde	A—[‘spicy’, ‘sweet’] B—[‘spicy’, ‘sweet’] C—[‘spicy’, ‘sweet’, ‘floral’]	*s*(*A*, *B*) > *s*(*A*, *C*)	*s*(*A*, *B*) < *s*(*A*, *C*)
3	A—4-Hydroxybenzaldehyde B—Coumarin C—Diethyl Succinate	A—[‘sweet’, ‘woody’] B—[‘sweet’] C—[‘fruit’, ‘floral’]	*s*(*A*, *B*) > *s*(*A*, *C*)	*s*(*A*, *B*) < *s*(*A*, *C*)
4	A—2,5-dimethyl pyrazine B—Ambrox C—Isopentyl acetate	A—[‘grass’, ‘woody’] B—[‘sweet’, ‘woody’] C—[‘sweet’, ‘fruit’]	*s*(*A*, *B*) > *s*(*A*, *C*)	*s*(*A*, *B*) < *s*(*A*, *C*)

Table 1 demonstrates that our model can correctly order the triplets where the distinction between similar (*A*, *B*) and dissimilar (*A*, *C*) odorant pairs is large. For instance, 4-Isopropylbenzyl alcohol smells quite distinct from benzyl disulfide as compared to ethyl cinnamate, and both expert and Deep metric predict the same similarity ordering. However, in the second example, the distinction between the smell impression of the odorant *A* and *C* is not significant, and our model’s prediction does not align with the expert similarity ordering. We believe that to achieve such fine-grained accuracy, models need to be trained on more diverse and larger dataset. We also observe that the mismatch between the expert and our model’s similarity ordering occurs more often when the dominant smell perception of odorants in the triplet is more specific such as “grass” or “woody” (third and fourth triplets in Table 1). The non-expert data lack such specificity in perceptual descriptors of odorants, and hence the learned model on non-expert data fails to accurately represent the perceptual dissimilarity. Overall, this indicates that our learned model can match with expert similarity assessment fairly well on easily distinguishable triplets and odorants with generic smell perception, familiar to non-expert users. However, better generalizability requires further investigation in several directions, which are a scope of future research—a) how to bring objectivity to the non-expert crowdsourced data, b) how to scale up model training on diverse data, and c) how to design the user’s study protocol to deduce the non-noisy data from non-experts.

## Conclusion

We presented several data-driven approaches to perceptual metric learning for estimating the human-perceived dissimilarity between molecules. First, we demonstrate the effectiveness of MDS in estimating perceptual dissimilarity. For generalizability on unseen test samples, we propose Mahalanobis and Deep metric learning methods that explicitly learn a parametric function over an input feature space and generalize easily to new unseen samples. Through extensive experiments, we show the effectiveness of various metrics in representing the perceptual similarity between odorants. We showed that perceptual features learned by deep embedding are effective in several learning-based tasks such as odorant retrieval, perceptual embedding, and relative/pairwise similarity estimation. We further demonstrate how odorants with distinct structural features and the functional group can still be perceptually similar, emphasizing the importance of incorporating user-specified perceptual similarity judgment in the modeling process. Finally, we demonstrate that the Deep metric trained on non-expert similarity feedback can well approximate the expert’s similarity assessment.

While our study shows promising results towards estimating perceptual dissimilarity between odorants, we believe that the model’s performance may be vulnerable to subjectivity in user responses. Additionally, the accuracy of the metrics can be further improved with more richer and informative structural features. Given the limited publicly available large database, we conducted an extensive study and evaluated our approach from various perspectives. There are several potential directions for future work. Moving forward, we would like to extend our study by developing an effective aggregation method to reliably deduce perceptual judgments from the crowdsourced data and evaluate the model trained on diverse data. To facilitate the evaluation of even larger datasets than those considered in this study, it will be interesting to investigate the utility of active learning for gathering selective user responses to learn effective perceptual metrics. Another promising direction to explore would be to build local metrics over data to address the subjectivity problem in user response.

## Supporting information

S1 Fig Performance of different metrics on pairwise distinguishability of molecules at low concentration. (TIF)

S2 Fig Perceptually similar odorants retrieved by Deep metric. The top-four perceptually similar odorants (in decreasing order from left to right) to Hydroxybenzaldehyde (top) and L-Cysteine (bottom) ranked by our learned model. For each query odorant, we show results of two models trained with and without “pleasant” descriptor.
(TIF)

S3 Fig Goodscents odorant’s distribution. Similar to the Keller dataset, the distribution of Goodscents descriptors is skewed. Few descriptors such as “sweet” and “green” are frequently used, and descriptors, such as “bitter” and “musk” are quite scarcely used. Moreover, there is a huge disparity between Goodscents and Keller descriptor sets. The goodscents data is annotated using 255 discrete descriptors, whereas the Keller dataset use only coarse 20 odor descriptors to describe 480 molecules. We consider common descriptors used in both datasets for our study. The common subset contains 16 descriptors—“sweet”, “woody”, “fruity”, “floral”, “chemical”, “fish”, “spicy”, “sour”, “sweaty”, “grass”, “acidic”, “ammonia”, “garlic”, “burnt”, “warm”, “musky”.
(TIF)

S4 Fig Performance of MDS as a function of dimension of the learned embedding space. We show the performance of MDS with increasing dimensions of embedding space. The left figure shows the value of the stress function, which indicates the difference between observed and estimated dissimilarity values of odorants. As expected, with increasing dimension, triplet generalization accuracy (right figure) improves as the learned features better represent the perceptual attributes.
(TIF)

S5 Fig Results shown for additional kernel-based metrics on Keller dataset for low (top) and high (below) concentrations. (TIF)
