# Supplementary figures and images for: Perceptual metrics for odorants: Learning from non-expert similarity feedback using machine learning
Source: PLoS One. 2023 Nov 8;18(11):e0291767. doi: 10.1371/journal.pone.0291767 (PMC10631653; doi:10.1371/journal.pone.0291767)

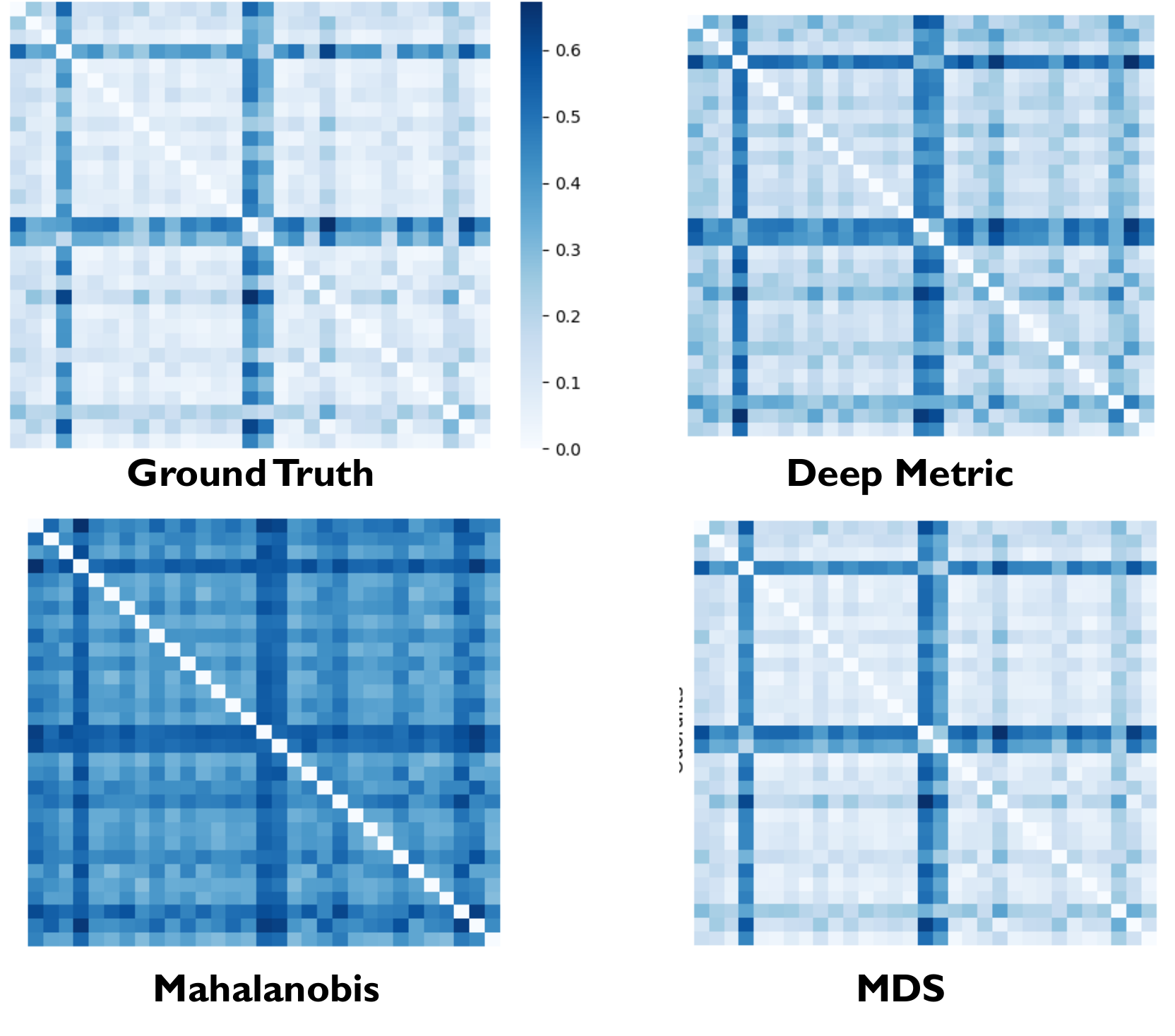

Supplement: S1 Fig — (TIF) [file pone.0291767.s001.tif]

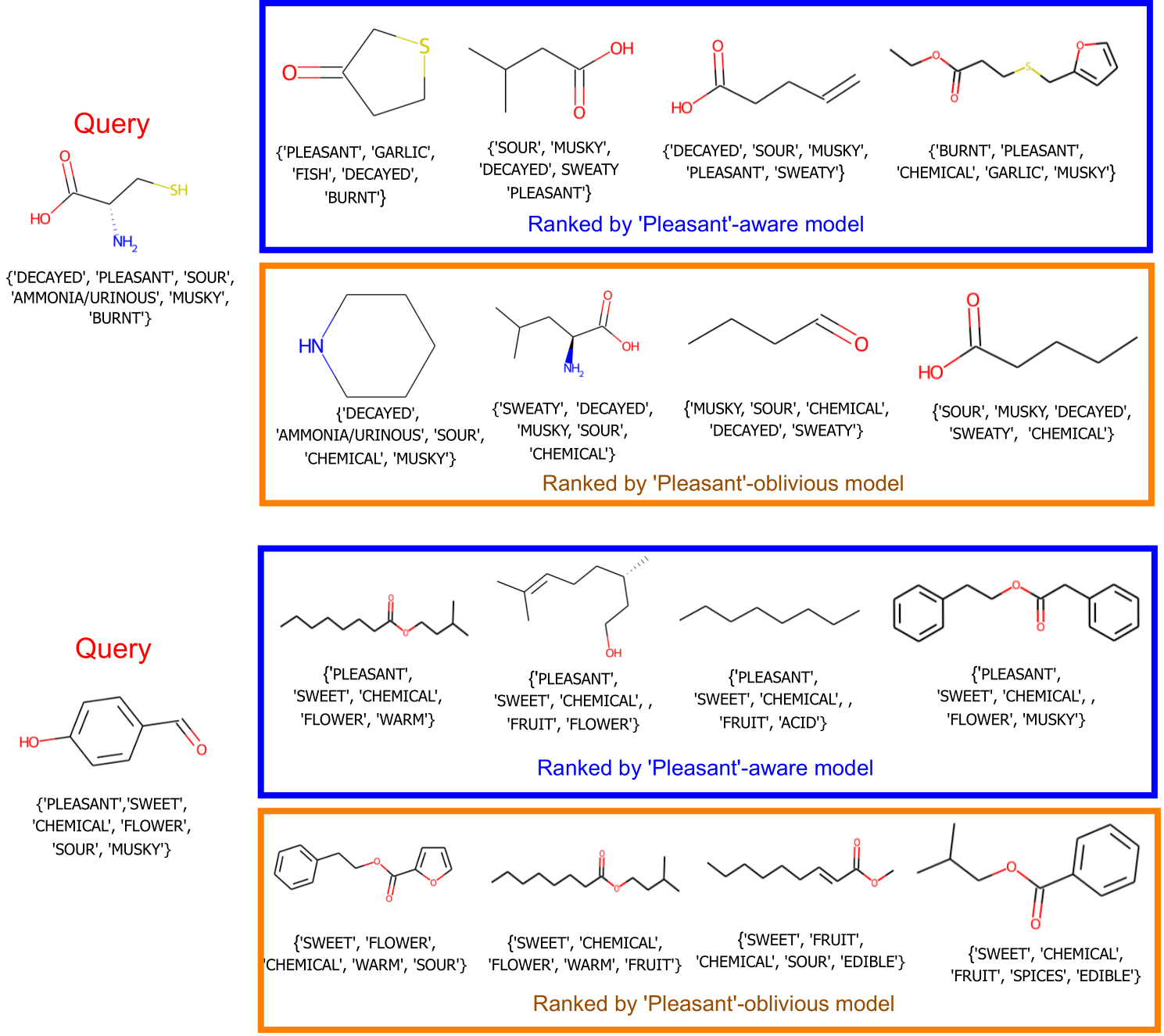

Supplement: S2 Fig — The top-four perceptually similar odorants (in decreasing order from left to right) to Hydroxybenzaldehyde (top) and L-Cysteine (bottom) ranked by our learned model. For each query odorant, we show results of two models trained with and without “pleasant” descriptor. (TIF) [file pone.0291767.s002.tif]

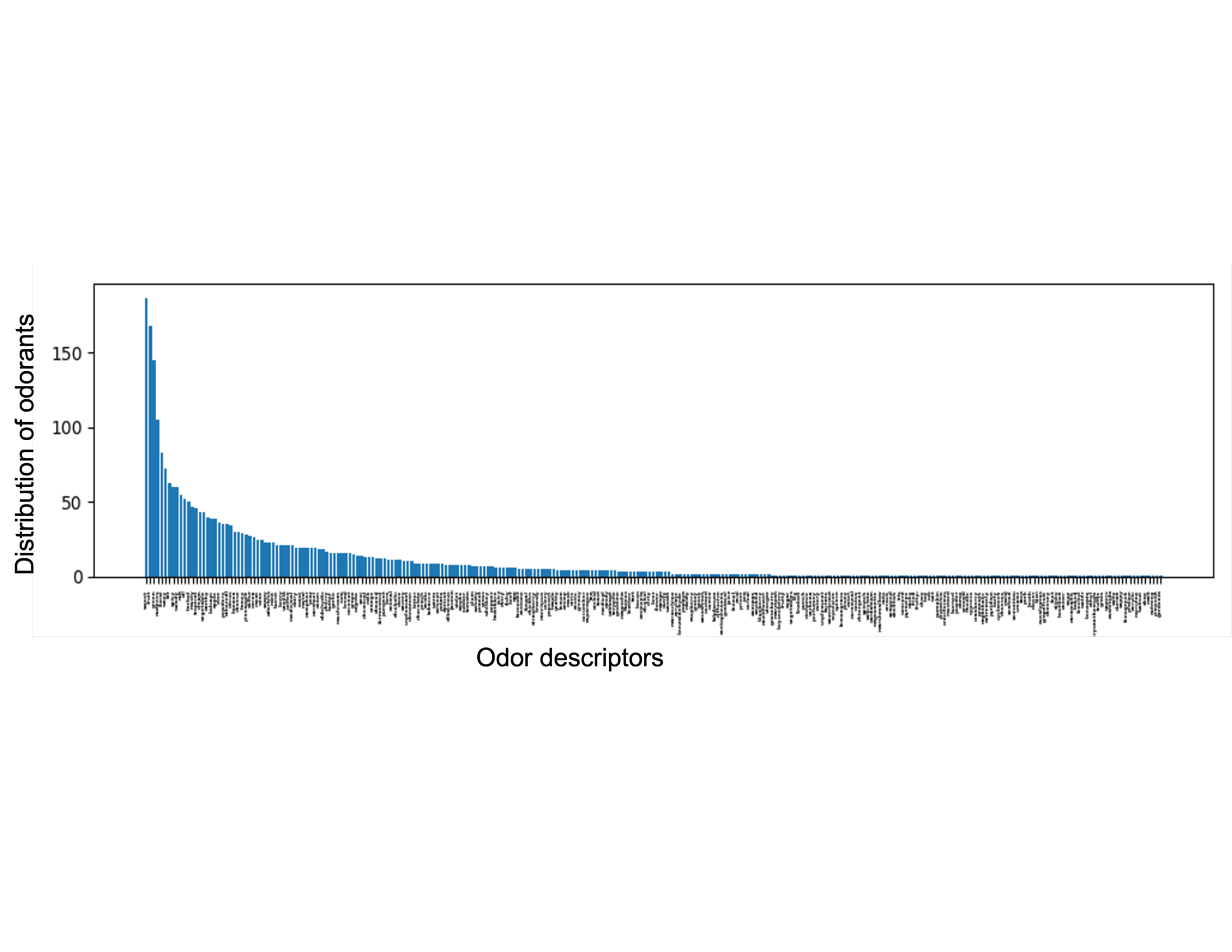

Supplement: S3 Fig — Similar to the Keller dataset, the distribution of Goodscents descriptors is skewed. Few descriptors such as “sweet” and “green” are frequently used, and descriptors, such as “bitter” and “musk” are quite scarcely used. Moreover, there is a huge disparity between Goodscents and Keller descriptor sets. The goodscents data is annotated using 255 discrete descriptors, whereas the Keller dataset use only coarse 20 odor descriptors to describe 480 molecules. We consider common descriptors used in both datasets for our study. The common subset contains 16 descriptors—“sweet”, “woody”, “fruity”, “floral”, “chemical”, “fish”, “spicy”, “sour”, “sweaty”, “grass”, “acidic”, “ammonia”, “garlic”, “burnt”, “warm”, “musky”. (TIF) [file pone.0291767.s003.tif]

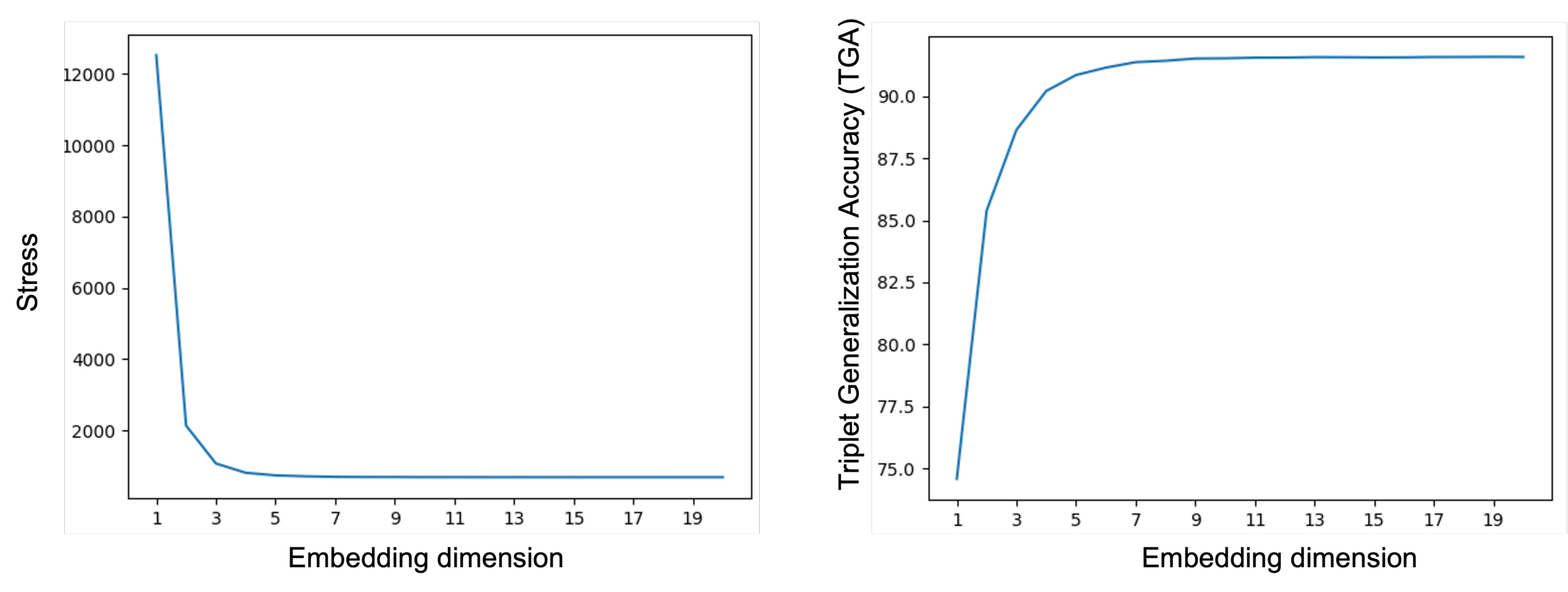

Supplement: S4 Fig — We show the performance of MDS with increasing dimensions of embedding space. The left figure shows the value of the stress function, which indicates the difference between observed and estimated dissimilarity values of odorants. As expected, with increasing dimension, triplet generalization accuracy (right figure) improves as the learned features better represent the perceptual attributes. (TIF) [file pone.0291767.s004.tif]

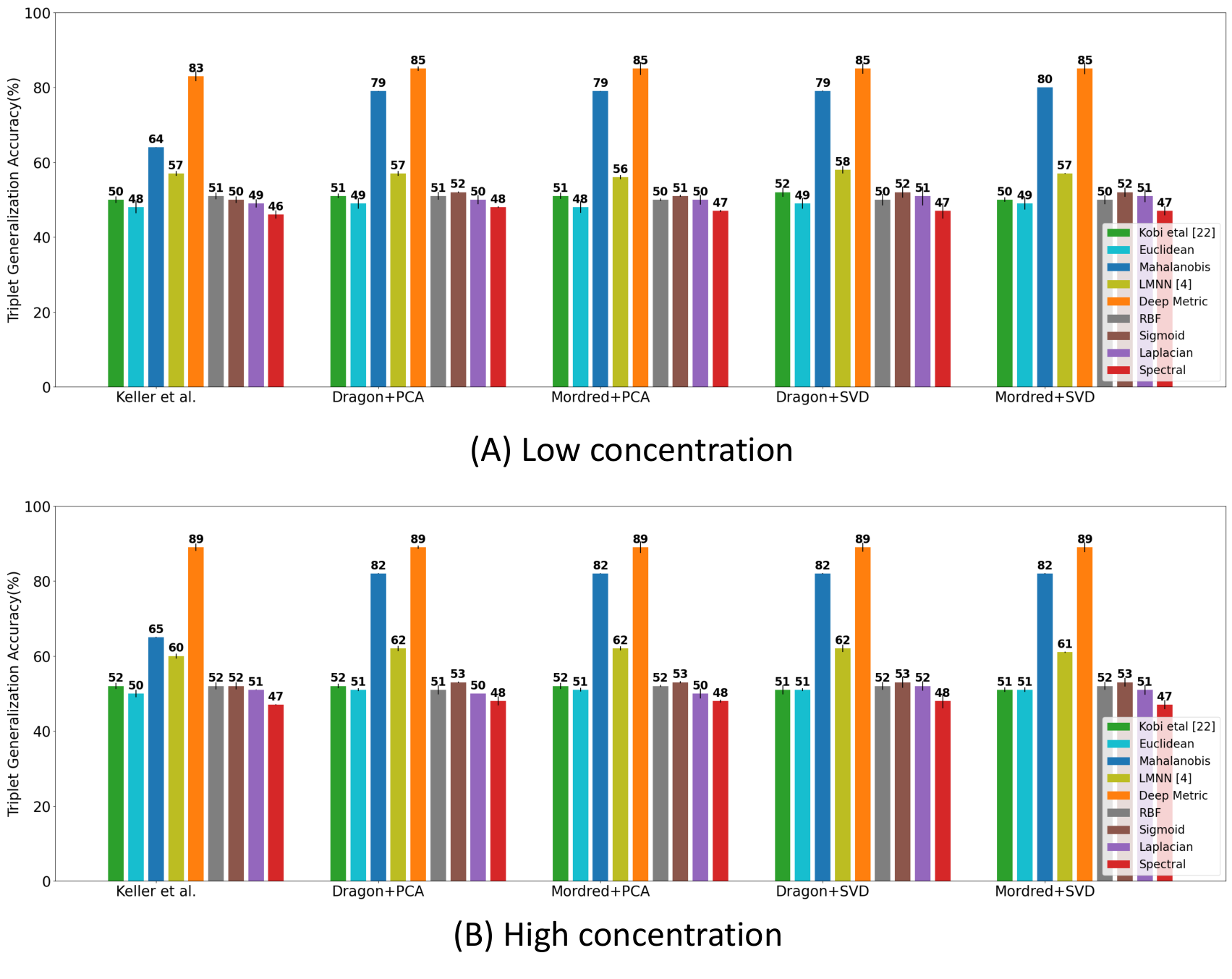

Supplement: S5 Fig — (TIF) [file pone.0291767.s005.tif]
